# Defining Potential Pathomechanisms Behind an Impaired Canal Function at the Video-Head Impulse Test in Canal Dehiscence. Reply to Ionescu et al. Comment on “Castellucci et al. Impaired Vestibulo-Ocular Reflex on Video Head Impulse Test in Superior Canal Dehiscence: “Spontaneous Plugging” or Endolymphatic Flow Dissipation? *Audiol. Res.* 2023, *13*, 802–820”

**DOI:** 10.3390/audiolres15020032

**Published:** 2025-03-17

**Authors:** Pasquale Malara, Salvatore Martellucci, Andrea Castellucci

**Affiliations:** 1 Audiology & Vestibology Service, Centromedico, 6500 Bellinzona, Switzerland; pasmalara@gmail.com; 2 ENT Unit, Santa Maria Goretti Hospital, Azienda USL di Latina, 04100 Latina, Italy; dott.martellucci@gmail.com; 3 ENT Unit, Department of Surgery, Azienda USL—IRCCS di Reggio Emilia, 42123 Reggio Emilia, Italy

We read with great interest the comment on our articles of Dr. Ionescu et al. [1]. We would like to thank the Authors for the consideration on our thoughts and for the pertinent observations they have advanced, with which we are largely in agreement. Moreover, we would like to thank the Authors, as they offered us the opportunity to clarify our precise position on the topic. A first preliminary but crucial point: as it is well known, superior canal dehiscence (SCD) syndrome can present with an extremely varied and ambiguous symptomatology that might induce clinicians to err, at least in some circumstances [2,3]. On the other hand, the likelihood that a vestibular disorder other than SCD could result in a selective hypofunction of the vestibulo-ocular reflex (VOR) gain for the superior semicircular canal (SSC) represents a rare event [4], contrary to what happens for the posterior semicircular canal (PSC) [5,6]. When the SSC is affected by a pathological process, the underlying pathomechanism usually involves other sensors of the inner ear (whether otolith or canal end-organs) and, even in these cases, the SSC represents the ampullary receptor exhibiting a tendency to recover sooner and better over time compared to other sensors [7,8,9,10]. Furthermore, contrary to other semicircular canals, it rarely seems to comply with a downregulation in the contest of a cerebellar clamping in the case of an injury affecting the functionally-coupled contralateral PSC (personal data representing an interesting topic and worthy of future investigation). This is the reason that led us to underline the potential interest in a selective deficit of the SSC at the video-head impulse test (vHIT) in various clinical investigations on canal dehiscence [11,12,13,14,15]. This data should not be understood as an element with an absolute diagnostic value, but rather as an indicator of the need to address the patient presenting with a selectively impaired SSC to additional tests with higher specificity and sensitivity for the diagnosis of SCD (i.e., the Tullio phenomenon and Hennebert sign, the skull vibration-induced nystagmus test (SVINT), and the vestibular-evoked myogenic potentials (VEMPs)), even though the clinical history is either atypical or is not fully consistent with a third window syndrome (TWS). We are therefore perfectly in agreement with the Authors of the comment when they pointed out that some cases with SCD, even with radiological evidence of probable spontaneous auto-plugging, can exhibit an SSC VOR-gain within normality ranges at the vHIT [1]. So true is it that a normal SSC VOR-gain has been detected even postoperatively in some patients submitted to surgical plugging, likely due to preserved cupular deflections after high-frequency head impulse, which is allowed by an endolymphatic redistribution within the remaining membranous canal [16,17,18,19]. Therefore, we totally agree with them in that the impaired SSC VOR-gain on vHIT cannot be considered a general rule for any natural evolution of an SCD. Nevertheless, we would like to underline, in general terms and not only in the case of possible SSC auto-plugging, how crucial is the high velocity when imparting head impulses in the plane of the presumed dehiscent canal [20,21,22]. For illustrative purposes, we enclose the case of a patient with left SCD exhibiting, besides low-frequency air-bone gap (ABG) and abnormally enhanced VEMPs on the left side (Figure 1A–D), a normal VOR-gain for the dehiscent SSC as long as the head peak velocity remains within 150°/s (i.e., a value which is considered absolutely consistent with the current recommendations on the use of the vHIT in the plane of vertical canals [23]) (Figure 1E), but broadly dropping to deficiency ranges (<0.7) when increasing the head impulse velocity over 200°/s (Figure 1F). Moreover, not only the dehiscent SSC is clearly impaired, unlike the contralateral SSC which is tested at the same velocity, but also a clear VOR-gain impairment for the functionally-coupled contralateral PSC can be detected (likely resulting from the loss of the push-pull mechanism), which is missing in the first vHIT traces with lower head velocities.

Despite all vHIT systems highlighting any deficit of the SSC, the capability of the device to offer the operator the real-time values of the imparted impulse velocity become crucial not to run into false negatives. This is because it is true that, as Authors already mentioned, some investigations showed that the VOR-gain of the affected canal at the vHIT could be normal even in canal dysplasia [24]. Nevertheless, those cases refer to canals embedded in an encased otic capsule, in which, thanks to the integrity of the bony labyrinth, the perilymphatic compartment is assumed to exert a cupular activation which is implemented through a process of mechanical deformation of the membranous canal structure, which supports and complements the deflection mechanism entrusted to the cupulo/endolymphatic dynamics (Figure 2A) [25,26]. On the contrary, in cases of SCD or other canal dehiscences, the mechanical role of the perilymphatic space is likely lacking and this support, which only occurs in the high-frequency domain, becomes either weaker or absent, leaving the canal VOR-gain value to the only activity guaranteed by the cupulo/endolymphatic dynamics (Figure 2B) [25,26]. This is why, in our opinion, the stimulation velocity is crucial in verifying the VOR-gain of a dehiscent SSC.

Like others, we also have wondered about the pathomechanisms underlying the selective deficit of the dehiscent canal at the vHIT. As Ionescu et al. have published [1,27], we also mentioned the hypothesis of a spontaneous auto-plugging process, either partial or intermittent, especially in those patients who presented with “atypical” symptoms and signs partly consistent either with TWS or with Meniere’s disease (MD) in addition to the selective high-frequency impairment for the affected SSC (Figure 2C) [11,12,13,14,15]. Nevertheless, the data of these patients, that might be affected by secondary dysfunctional phenomena, were deliberately excluded from our research, as our aim was to concentrate our reasoning exclusively on “typical” SCD with “pure” TWS signs and symptoms [14]. In fact, in this latter paper, we aimed to propose to go beyond the concept of a spontaneous auto-plugging as the cause of the SSC VOR-gain, and we introduced a different and alternative idea: the dissipation of the ampullofugal endolymphatic flow during the head impulses (Figure 2B) [13,14]. However, in some situations, these two conditions could coexist. Theoretically, the pathomechanism of a spontaneous partial auto-plugging could certainly result in a transient shift of the endolymphatic volume from the SSC to the nearby portions of the labyrinth, thus determining a condition consistent with a secondary endolymphatic hydrops (EH) which might also be clearly visible in MRI with a dedicated protocol. This condition might lead to symptoms consistent with MD or atypical symptoms for SCD. We enclose a clinical case of a 55-year-old lady exhibiting such a condition, not only in imaging, but also in symptoms and signs consistent with left MD (Figure 3).

On the contrary, the alternative concept of an endolymphatic flow dissipation as a cause of the VOR-gain impairment for the dehiscent SSC, which has been extensively explained in all our papers, came up from several considerations. The first is that, when comparing instrumental signs of patients with surgically plugged SCD to a group of SCD patients who did not undergo surgery, we observed significant differences in VEMPs and ABG in our investigation, while the VOR-gain for the dehiscent SSC at the vHIT was roughly similar in both groups. In particular, VEMPs amplitudes significantly reduced and thresholds increased in patients who were operated on, while VEMPs were overall enhanced in the non-surgical group. Similarly, the ABG of most of the patients who underwent surgery reduced, while most of the patients from the non-surgical group exhibited a significant ABG [14]. In light of these results, we concluded that it is less likely that the canal VOR-gain deficit in SCD patients presenting with TWS can be ascribed to a natural plugging. Conversely, a dissipation of endolymphatic flows at the dehiscence during vertical head impulses could likely account for the impaired SSC function at the vHIT. Secondly, we realized how the high-velocity VOR-gain of some dehiscent PSC was impaired even though the anatomic position of the PSC precludes a plugging exerted by the surrounding structures. Only in one case could the PSC impairment be attributed to a herniation of the jugular bulb through the PSC dehiscence [15]. These data could suggest that, in some circumstances, a selective deficit for the dehiscent canal may be ascribed to dynamics other than a spontaneous auto-plugging process.

Another consideration: analyzing some studies on animal models, the increased VEMPs responses in patients with SCD are probably due to the joint action of the irregular afferents of both the dehiscent SSC and the otolithic organs, which share the same ocular muscle targets and which can be activated only in the case of dehiscence, while remaining silent (as regard to the canal afferents) with an encased otic capsule [28,29,30,31]. The clinical case of Figure 3 represents an example of the coexistence between radiological and clinical/instrumental signs of both EH and SCD. Here, the SVINT, the vHIT, and tone burst stimuli via air-conduction (AC) were used to verify in humans the accuracy of this hypothesis obtained through electrophysiological recordings in guinea pigs [30]. Downbeating/left-torsional nystagmus aligning with the plane of the dehiscent SSC was clearly detected during the SVINT and in response to loud sounds (Video S1). On the other hand, during transient AC stimuli with 2–4 kHz tone bursts, we documented an upbeating ocular movement which was phase-locked with the frequency of the tone burst stimulation (Video S1). This ocular movement immediately ceased at the end of the transient AC stimulus that was triggering it. We believe that this oculomotor finding (i.e., the slow phase of a downbeating nystagmus that never occurs due to the transient endolymphatic flow generated by the tone bursts) resulted from the transient activation of the irregular afferents of the dehiscent SSC exhibiting an hyperexcitability due to the dehiscence itself. The resulting VEMPs clearly exhibited enhanced amplitudes and lowered thresholds, and the fact that the aforementioned ocular movements were also detected in response to 4 kHz AC-stimuli suggests that the irregular canal afferents also contributed to the enhanced VEMPs in response to very high-frequency stimuli [32,33]. This case, in which an associated EH was clearly visible on the MRI alongside the SCD on the same ear, highlights in our opinion how a spontaneous canal plugging, which may have generated a secondary EH in the inner ear, does not have the same effectiveness as surgical plugging, as we have clearly emphasized in our papers [13,14]. In fact, the endolymphatic flows produced by sound and pressure stimuli are likely strong enough to overcome the dural plugging and generate abnormal stimulation of the canal afferents.

On the other hand, in our cohort of patients with SCD, some subjects with typical symptoms and signs consistent with a TWS showed nystagmus aligning with a purely horizontal plane and long-lasting post-stimulatory reverberance at the SVINT [34], similar ocular movements in response to sounds/pressure stimuli, and a significant VOR-gain impairment for the affected SSC at the vHIT (unpublished data), as if the dehiscent SSC afferents could not be activated neither in the high- nor in the low-frequency domain [35]. Nevertheless, both cervical and ocular VEMPs were still clearly enhanced even to high-frequency AC stimuli. For illustrative purposes, we enclose the case of a 74-year-old patient with left SCD exhibiting, besides low-frequency air-bone gap (ABG) and enhanced VEMPs on the left side, a reduced VOR-gain for the left SSC along with an impairment for both PSC (Figure 4), consistent with a compound action of left SCD and age-related bilateral vestibulopathy [36]. The same patient presented with purely horizontal nystagmus induced by SVINT and loud sounds (Video S2). These data reinforced the idea we advanced in our papers, i.e., that the VOR-gain impairment for the dehiscent SSC at the vHIT might be more easily due to a dissipation process for the endolymphatic flows rather than to a spontaneous canal plugging. In fact, the same dissipation process accounts for the enhanced VEMPs responses and for the ABG in audiometry, consistent with a TWS [3].

In conclusion, further studies in human and animal models are needed to fully understand the pathomechanisms behind the functional impairment of the effected canal in patients with SCD and other canal dehiscences presenting either with symptoms consistent with a TWS or with atypical profiles.

## Figures and Tables

**Figure 1:**
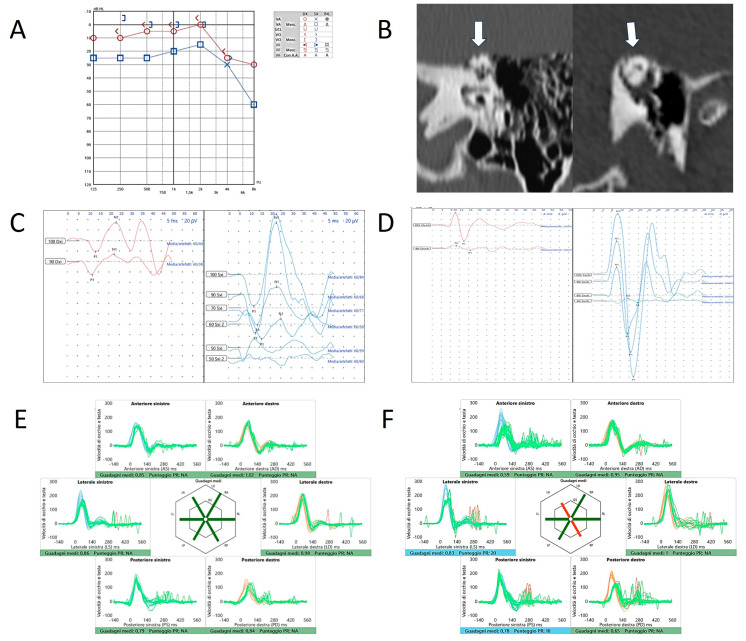
Instrumental and radiological data in patient n.1 (a 57-year-old male with left-sided SCD). Audiometry (**A**) showing low-frequency ABG and HRCT (**B**) with coronal scans and parasagittal reconstructions showing SCD on left side (white arrows). Cervical (**C**) and ocular-VEMPs (**D**) exhibiting low thresholds and high amplitudes on the left side. (**E**) vHIT performed with standard head velocities (150°/s) showing normal VOR-gain values for all the canals. (**F**) vHIT performed with higher head velocities (250°/s) showing reduced VOR-gain values for the left SSC and the right PSC. Blue lines represent head impulses exciting left canals, orange lines correspond to impulses for right canals, green lines represent eye movements induced by the activation of the VOR following each impulse and red lines correspond to corrective saccades. Mean value of VOR-gain (eye velocity/head velocity) is reported for each canal. The hexagonal plot in the center of the figure summarizes mean VOR-gains for each canal; normal gains are shown in green and deficient gains are in red. Gains are considered as normal if more than 0.8 for lateral canals and more than 0.7 for vertical canals.

**Figure 2:**
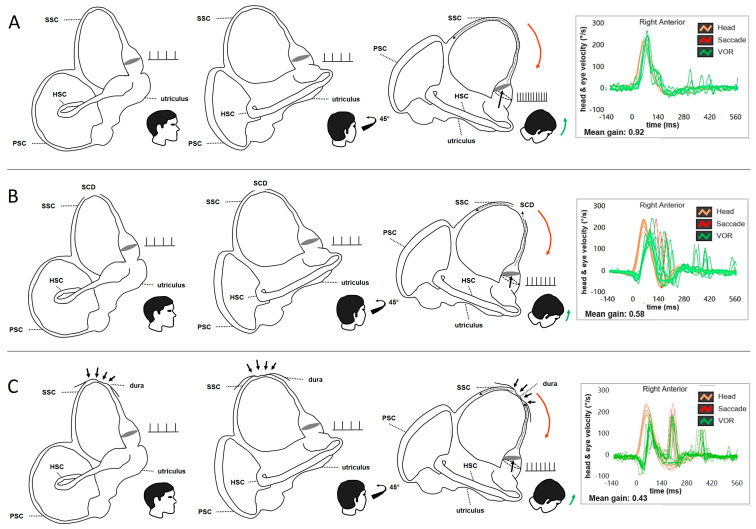
Schematic drawing of a right-sided labyrinth at rest and during downward head impulses along the plane of the tested SSC (RALP) with the representation of the firing rates of the SSC ampullary afferents and the corresponding SSC VOR-gain at the vHIT (modified from Castellucci et al. [13]). There are three different scenarios: (**A**) Normally encased SSC. During downward head impulses, the endolymphatic flows are correctively driven upward along the tested SSC (black dotted arrow) due to the support of the overlying perilymphatic fluid which maximally pulls ampullofugally the cupula from its original resting position (black dashed circle). In this physiological condition, the ampullary afferents are maximally excited, leading to a maximal increase in the resting firing rate. Orange lines and arrows correspond to downward head impulses for the right SSC and green lines and arrows represent upward eye movements induced by the activation of the VOR following each impulse. The mean VOR-gain value for the tested SSC settles within normal limits (0.92). (**B**) Endolymphatic flow dissipation in SCD. During downward head impulses, the action of the overlying perilymphatic space is lacking and part of the fluid-mechanical wave is wasted at the dehiscence (black dotted arrows), reducing the amount of endolymphatic flow efficiently pulling the cupula ampullofugally. Therefore, the cupula is only partly displaced, increasing to a lesser extent the resting firing rate of the ampullary afferents. An impaired mean value of VOR-gain (0.58) is therefore detected. (**C**) Natural incomplete canal plugging in SCD. The overlying middle fossa dura herniates in part within the SSC leading to an incomplete plugging of the canal (black arrows). During downward head impulses, the partial occlusion of the canal reduces the amount of endolymphatic flow efficiently pulling the cupula ampullofugally. Therefore, the cupula is less displaced, increasing to a lesser extent the resting firing rate of the SSC ampullary afferents, leading to an impaired VOR-gain (0.43).

**Figure 3:**
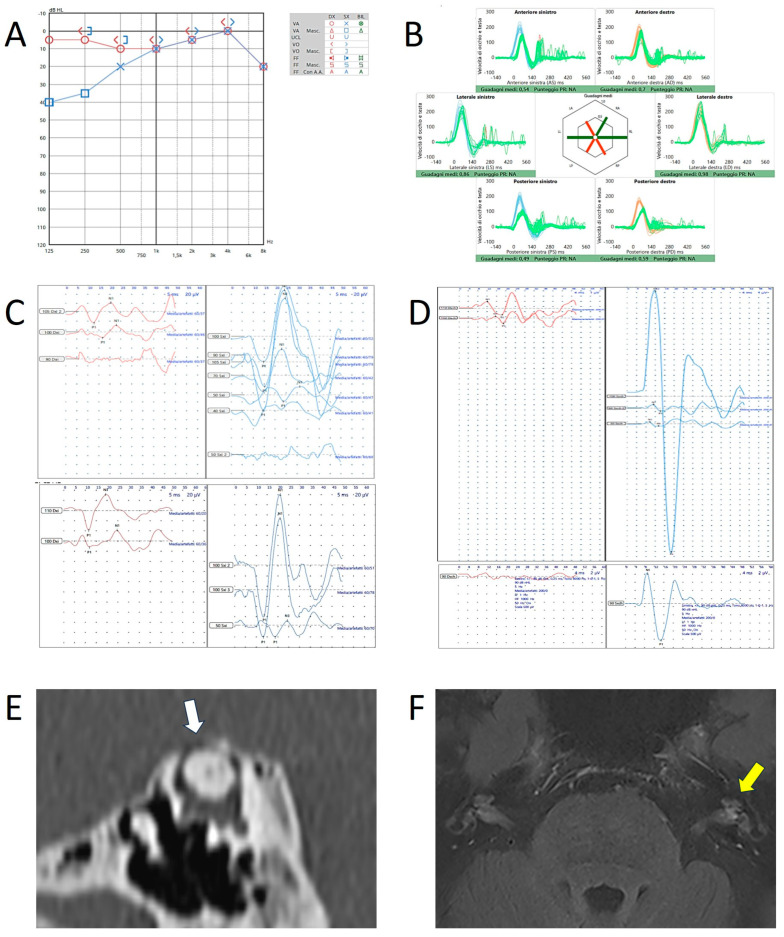
Instrumental and radiological data in patient n.2 (a 55-year-old female with left-sided SCD and ipsilateral MD). (**A**) Audiometry showing low-frequency ABG in the left side. (**B**) vHIT showing reduced VOR-gain values for both SSC and PSC on the left side and reduced values for contralateral PSC likely due to the lack of the push–pull mechanism during head impulses on the LARP plane. Cervical- (**C**) and ocular-VEMPs (**D**) exhibiting low thresholds, high amplitudes, and frequency tuning on the left side. In both cases, the upper image refers to potentials in responses to 500 Hz tone bursts while the lower panel refers to potentials in response to 1 kHz tone burst for cervical-VEMPs and to potentials in response to 8 kHz tone bursts for ocular-VEMPs. (**E**) HRCT showing left SCD (white arrow). (**F**) 3T brain-MRI with delayed acquisition following Gadolinium administration detecting signs of EH in the left labyrinth (yellow arrow).

**Figure 4:**
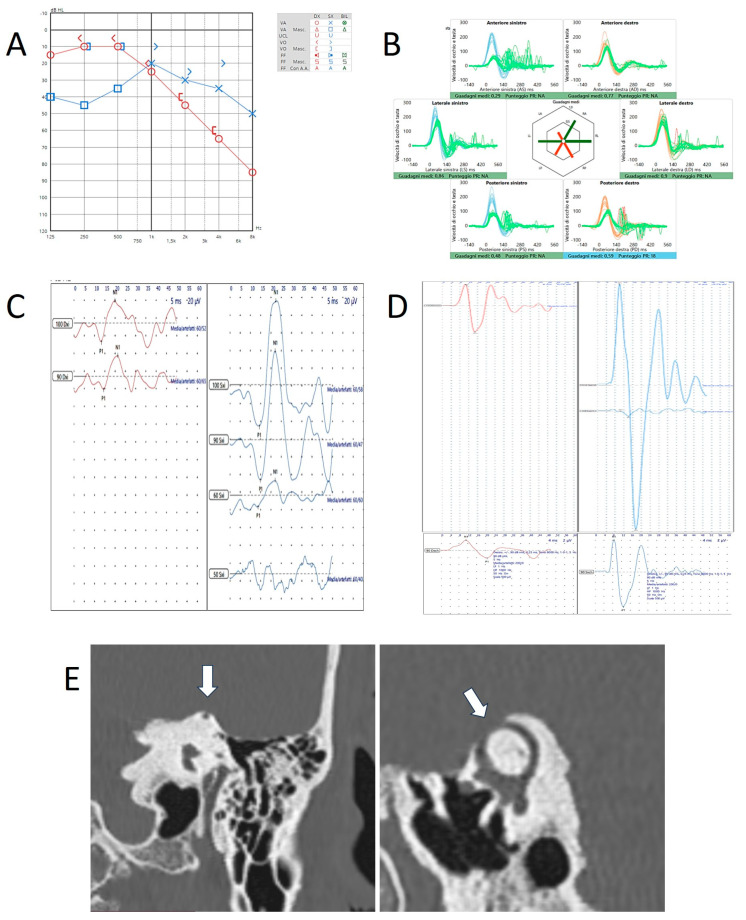
Instrumental and radiological data in patient n.3 (a 74-year-old patient with left-sided SCD). (**A**) Audiometry showing low-frequency ABG in the left side. (**B**) vHIT showing reduced VOR-gain values for the left SSC and both PSC. Cervical- (**C**) and ocular-VEMPs (**D**) exhibiting low thresholds and high amplitudes on the left side. The lower panel refers to ocular potentials in response to 8 kHz tone bursts. (**E**) HRCT scan with coronal and parasagittal reconstructions showing left SCD (white arrows).

## Supplementary Materials

The following supporting information can be downloaded at: https://www.mdpi.com/article/10.3390/audiolres15020032/s1, Video S1: Video-Frenzel recording of patient n. 2. Video S2: Video-Frenzel recording of patient n. 3.

## Abbreviations

The following abbreviations are used in this manuscript:

ABG	Air-bon gap
AC	Air-conduction
EH	Endolymphatic hydrops
HRCT	High resolution CT
HSC	Horizontal semicircular canal
LARP	Left anterior—right posterior
MD	Meniere’s disease
MRI	Magnetic resonance imaging
PSC	Posterior semicircular canal
RALP	Right anterior—left posterior
SCD	Superior canal dehiscence
SSC	Superior semicircular canal
SVINT	Skull vibration-induced nystagmus test
TWS	Third window syndrome
VEMPs	Vestibular-evoked myogenic potentials
vHIT	Video-head impulse test
VOR	Vestibulo-ocular reflex
